# Identification of Novel Quantitative Trait Loci Linked to Crown Rot Resistance in Spring Wheat

**DOI:** 10.3390/ijms19092666

**Published:** 2018-09-08

**Authors:** Gul Erginbas-Orakci, Deepmala Sehgal, Quahir Sohail, Francis Ogbonnaya, Susanne Dreisigacker, Shree R. Pariyar, Abdelfattah A. Dababat

**Affiliations:** 1 Global Wheat Program, International Maize and Wheat Improvement Center (CIMMYT), Ankara 06511, Turkey; G.Erginbas@cgiar.org; 2 International Maize and Wheat Improvement Center (CIMMYT), Apdo. Postal 6-641, Mexico DF06600, Mexico; D.Sehgal@cgiar.org (D.S.); S.Dreisigacker@cgiar.org (S.D.); 3 International Winter Wheat Improvement Program (IWWIP), International Maize and Wheat Improvement Center (CIMMYT), Ankara 06511, Turkey; quahirsohail@hotmail.com; 4 Institute of Biotechnology and Genetic Engineering, The University of Agriculture Peshawar, Peshawar 25000, Pakistan; 5 Grains Research and Development Corporation (GRDC), P.O. Box 5367, Kingston, ACT 2604, Australia; Francis.Ogbonnaya@grdc.com.au; 6 Institute of Bio- and Geosciences, Plant Sciences (IBG-2), Forschungszentrum Jülich GmbH, 52425 Jülich, Germany; s.pariyar@fz-juelich.de

**Keywords:** *Fusarium culmorum*, crown rot resistance, genome-wide association study, structure, quantitative trait loci, *Triticum aestivum*

## Abstract

Crown rot (CR), caused by various *Fusarium* species, is a major disease in many cereal-growing regions worldwide. *Fusarium culmorum* is one of the most important species, which can cause significant yield losses in wheat. A set of 126 advanced International Maize and Wheat Improvement Center (CIMMYT) spring bread wheat lines were phenotyped against CR for field crown, greenhouse crown and stem, and growth room crown resistance scores. Of these, 107 lines were genotyped using Diversity Array Technology (DArT) markers to identify quantitative trait loci linked to CR resistance by genome-wide association study. Results of the population structure analysis grouped the accessions into three sub-groups. Genome wide linkage disequilibrium was large and declined on average within 20 cM (centi-Morgan) in the panel. General linear model (GLM), mixed linear model (MLM), and naïve models were tested for each CR score and the best model was selected based on quarantine-quarantine plots. Three marker-trait associations (MTAs) were identified linked to CR resistance; two of these on chromosome 3B were associated with field crown scores, each explaining 11.4% of the phenotypic variation and the third MTA on chromosome 2D was associated with greenhouse stem score and explained 11.6% of the phenotypic variation. Together, these newly identified loci provide opportunity for wheat breeders to exploit in enhancing CR resistance via marker-assisted selection or deployment in genomic selection in wheat breeding programs.

## 1. Introduction

Bread wheat (*Triticum aestivum* L.) is one of the most important cereal crops. It is an allohexaploid (2*n* = 6*x* = 42, AABBDD) originated from the result of a rare natural hybridization between tetraploid emmer wheat (AABB, *T. dicoccoides*) and the diploid wild goat grass (DD, *Aegilops tauschii*) around 8000 to 10,000 years ago [1,2,3,4,5]. As a staple food, it provides nearly 55% of the carbohydrates and 20% of the food calories consumed globally [6]. Turkey is among the top 10 wheat producers worldwide and produces 16 to 21 million tons annually, of which 90% is grown under rainfed or semi-arid conditions [7]. Crown rot (CR)—caused by various Fusarium species—is one of the most damaging diseases of wheat [8]. CR is mostly caused by *Fusarium pseudograminearum*, but *F. culmorum* has also been shown to cause significant reductions in wheat yields, thus both species are considered economically important [8,9]. CR has been reported in West Asia (Turkey, Iraq, and Iran), North Africa (Egypt, Tunisia, and Morocco), United States, Canada, and Australia [8,9,10,11,12]. Yield losses of up to 43%, 45.5%, and 61% have been reported in Turkey [10], Iran [11] and the US [13,14], respectively. The extent of the damage depends on environmental conditions, agronomic and farming system management practices, and the virulence of the fungal population [14]. CR is particularly prevalent under drought and rainfed conditions, and in wheat monoculture systems [15,16].

Use of resistant cultivars is the most effective way of managing CR and ultimately improving crop productivity, especially in dry areas. Despite significant research efforts, only a limited number of varieties with partial resistance have been identified to date which can be attributed to the disease complexity coupled with the fact that the host resistance to CR is not pathogen species-specific [17]. Molecular markers associated with CR resistance could therefore be useful in incorporating diverse sources of resistance in elite germplasm. Genome-wide association study (GWAS) can be used to identify quantitative trait loci (QTL) in large sets of diverse populations which may be amenable for deployment in marker assisted selection for genome wide selection. GWAS utilizes linkage disequilibrium to dissect the genetic architecture of complex traits by correlating phenotypes to genotypes [18,19]. It has been utilized successfully in wheat to identify QTL linked to many important agronomic traits such as flowering time, plant height, grain yield, milling quality and disease resistance [18,20,21,22,23,24,25,26,27,28].

QTLs for resistance to CR pathogens have been identified previously using double haploid (DH) lines. Wallwork et al. (2004) assessed 100 DH lines from a cross between Australian cultivars Kukri (moderately resistant, MR) and Janz (susceptible, S) in an outdoor terrace system inoculated with *F. pseudograminearum* and *F. culmorum.* They identified a QTL on chromosome 4B, linked to the dwarfing gene *Rht-B1* [29]. Similarly, Collard et al. (2005) conducted a seedling-based phenotypic screening under glasshouse conditions of 145 DH lines derived from a cross between cultivars 2–49 (MR) and Janz [30]. They identified two QTLs on chromosomes 1D and 1A that explained 21% and 10% of phenotypic variance, respectively. Another study identified a QTL on chromosome 5D explaining 10.2% of phenotypic variance [31] from a cross between cultivars W21MMT70 (MR) and Mendos (MS). More recently, QTL conferring resistance to CR have been reported on chromosome 3BL [32,33,34], which are effective against both *F. graminearum* and *F. pseudograminearum.* Despite these reports, no variety with adequate level of resistance to *F. culmorum* is currently available and research on genomic regions associated with resistance to *F. culmorum* has received no attention. Recently, a candidate gene-based association study reported association of mitogen-activated protein kinase (MAPK) *HOG1* gene with aggressiveness and deoxynivalenol (DON) production, explaining 10.29 and 6.05% of the genotypic variance, respectively [35]. This present study aims to improve resistance of spring bread wheat to *F. culmorum* with the following objectives: (i) analyze resistance responses of spring bread wheat accessions to *F. culmorum*, and (ii) use GWAS to identify novel genomic loci conferring resistance to *F. culmorum*.

## 2.Results

### 2.1. Fusarium culmorum Disease Assessment

**Figure 1:**
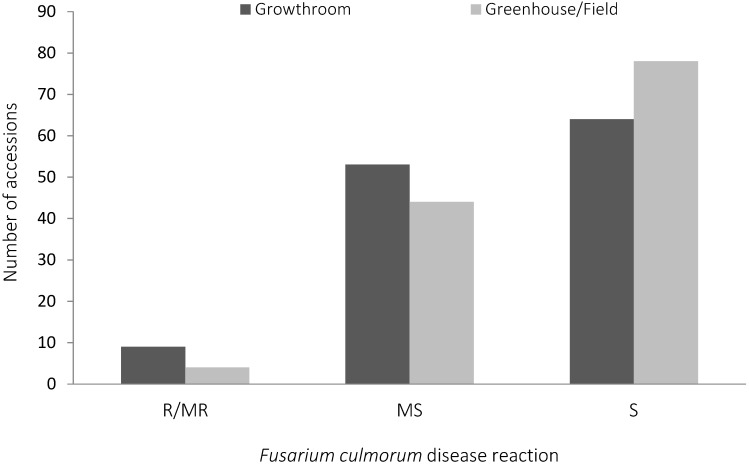
Resistance reactions of 126 advanced CIMMYT spring bread wheat accessions to crown rot (*Fusarium culmorum*). R = resistant, MR = moderately resistant, MS = moderately susceptible, and S = susceptible under growth room (seedling resistant), greenhouse, and field conditions (adult plant resistant).

The 126 spring bread wheat accessions were classified into three groups (resistant to susceptible) according to their CR resistant reactions under growth room, greenhouse, and field conditions (Figure 1, Table S1). When seedlings were screened under growth room conditions for resistance to *F. culmorum*, 3% of the spring wheat accessions were resistant to moderately resistant (R-MR), 35% were moderately susceptible (MS), and 62% were susceptible (S). When tested for adult plant resistance under greenhouse and field conditions, 7% were R-MR, 42% were MS, and 51% were S. The eight wheat cultivars used as controls in the screening were consistent across experiments, in terms of their response to *F. culmorum* (Table 1). Spring bread wheat cultivars 2–49 and Sunco and the winter wheat cultivar Altay-2000 showed the maximum disease reduction. The tetraploid wheat cultivar Kiziltan-98 displayed higher disease expression than the known susceptible wheat cultivars Seri-82, Wylie, and Janz. Broad sense heritabilities for the different traits were; 0.4379, 0.1708, 0.3696 and 0.4725 for the greenhouse crown score, the field crown score, growth room crown score, and for the greenhouse stem score, respectively. The correlation between two pairs of traits was significant and positive; (a) field crown score and growth room crown score and (b) between greenhouse crown scores and greenhouse stem scores (Table 2).

### 2.2. Genotyping with Diversity Arrays Technology (DArT)Markers

**Figure 2:**
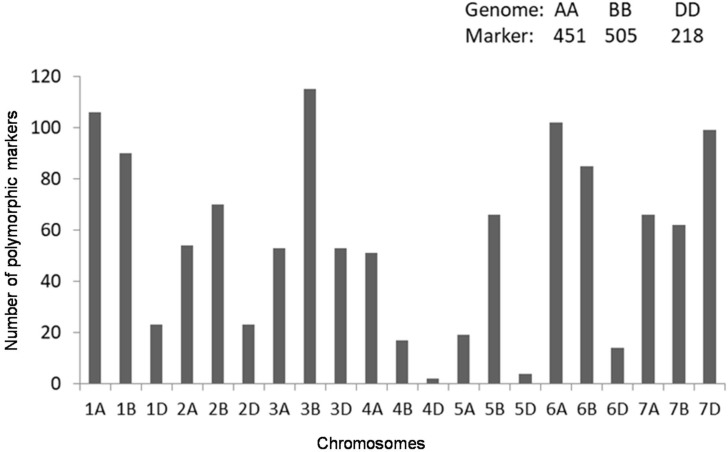
Distribution of the Diversity Array Technology (DArT) markers on the 21 chromosomes of the 107 advanced CIMMYT spring bread wheat accessions.

The 107 accessions were genotyped with high-density DArT markers, which produced 1726 polymorphic markers across all accessions. Of these, 1174 filtered markers (with known map positions, marker allele frequency >0.05 and <0.95 and max. 20% missing data) were used for further analysis. In total, 451, 505, and 218 markers were mapped on the A, B, and D genomes, respectively (Figure 2).

### 2.3. Structure Analysis

**Figure 3:**
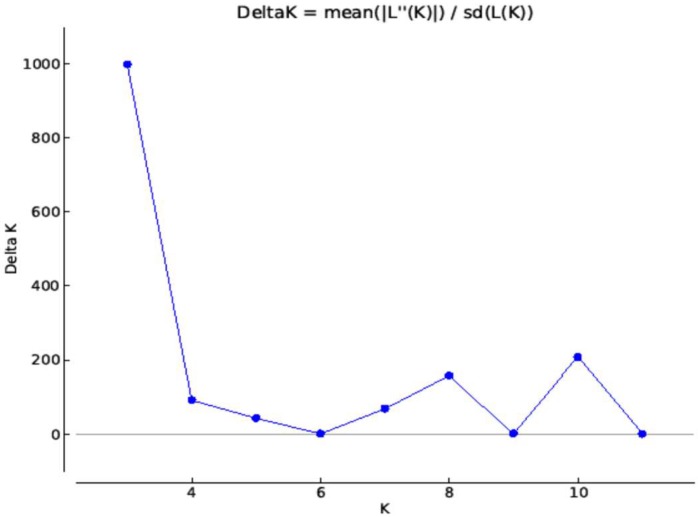
Average logarithm of the probability of data likelihood [Ln P(D)]; Ad-hoc statistic ∆K for K values ranging from 1 to 12.

**Figure 4:**
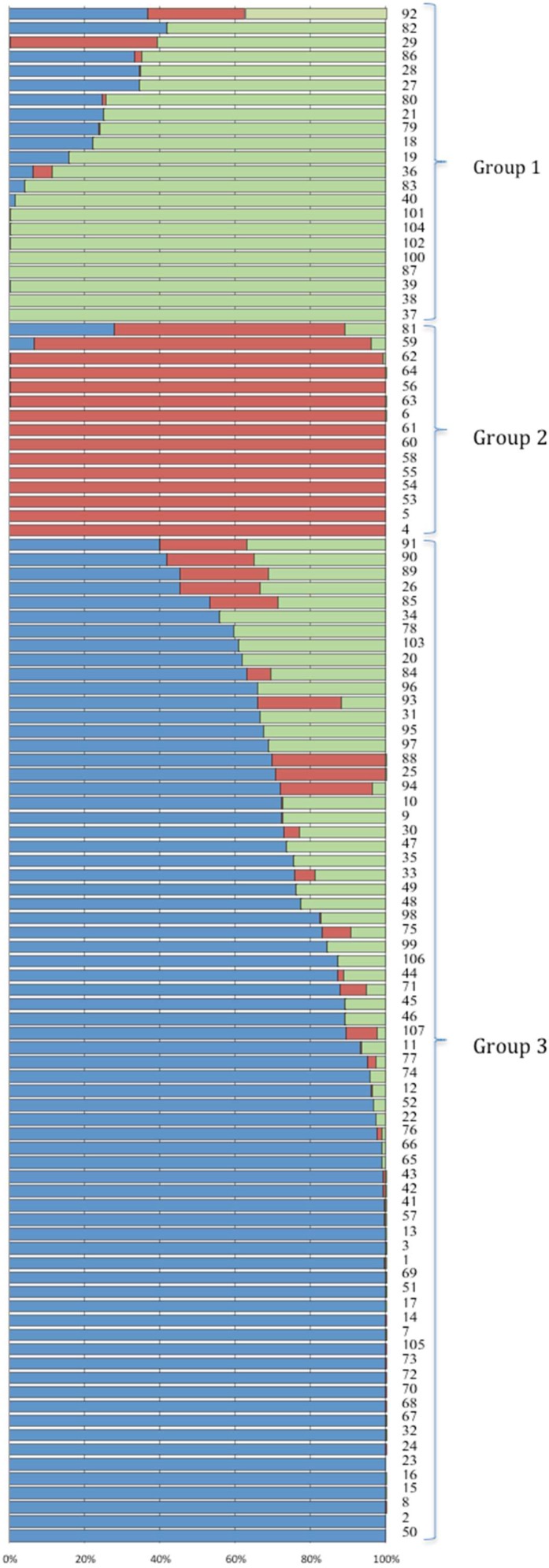
Population structure of 107 advanced CIMMYT spring bread wheat accessions constructed by using 1174 DArT polymorphic markers. Three subpopulations represented by green, red and blue colors are shown at K = 3. Each accession (wheat line) is represented by a thin vertical line (bar), which can be partitioned into three colored segments representing estimated membership probabilities (Q) of the individual.

Results from Delta K vs. K plot (Figure 3) suggested three sub-populations in the panel used. The principal component analysis (PCA) plot (Supplementary Figure S1) also showed three groups. Therefore, K = 3 best explained groupings in the panel (Figure 4). The first sub-group consists of 22 accessions; all genotypes had Sokoll in their pedigrees except lines 29 and 79. The second group consisted of 15 genotypes. Six genotypes (5, 6, 61, 62, 63 and 64) in this group were sister lines. The third group was the biggest group and consisted of 70 genotypes. This group was comparatively more diverse, although there was no particular parent or line, which was present in the majority of the lines, but around 34 genotypes had BAV92 in their pedigree, 29 had Pastor, 25 had Milan and 22 had Prinia in their pedigrees.

### 2.4. Linkage Disequilibrium

**Figure 5:**
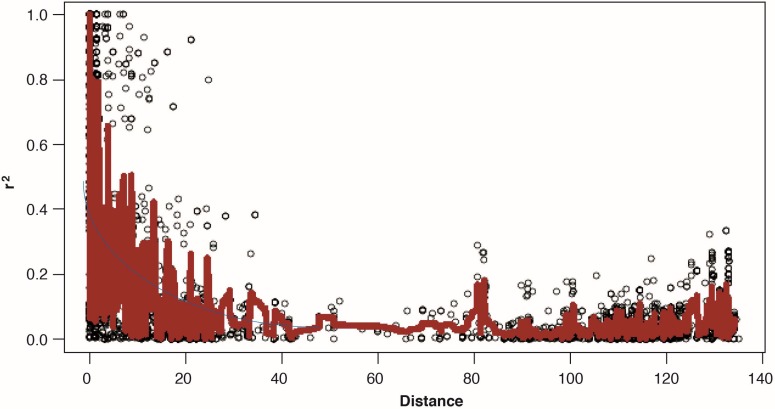
Scatterplot showing linkage disequilibrium (LD) decay estimated by (*r*^2^) against genetic distance (cM) in 107 advanced CIMMYT spring bread wheat accessions. The blue curve is the LOESS approximation of mean LD.

Linkage disequilibrium (LD) was estimated by calculating squared correlation coefficient (*r*^2^) for all 36,654 marker pairs (ranging from 91 marker pairs on chromosome 6D to 4475 marker pairs on chromosome 3B). Genome-wide, LD decayed rapidly with genetic distance and it reached to 50% of its initial value at around eight centi-Morgan (cM) and reached below cut off *r*^2^ = 0.1 at around 20 cM (Figure 5). Table S2 shows chromosome wise LD for all chromosomes except chromosome 4D and 5D, for which LD was not calculated due to scanty markers.

### 2.5. Marker-Trait Associations for F. culmorum Resistance

**Figure 6:**
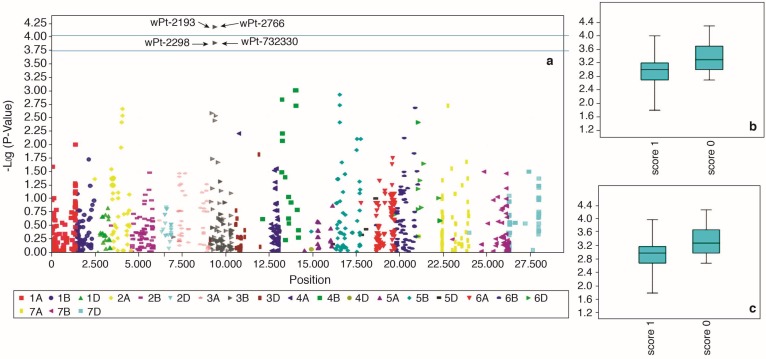
Manhattan plot showing association of 3B markers with CR resistance using field crown scores (**a**). The two markers wPt-2193 and wPt-2766 with exactly same *p* value are represented as a single dark grey spot above threshold line at 4.00. These markers crossed false discovery rate at *p* < 0.05. The markers wPt-2298 and wPt-732330 are in LD with wPt-2193 and wPt-2766 and show exactly the same *p* value but at a lower threshold. Allelic effects of wPt-2193 (**b**) and wPt-2766 (**c**).

**Figure 7:**
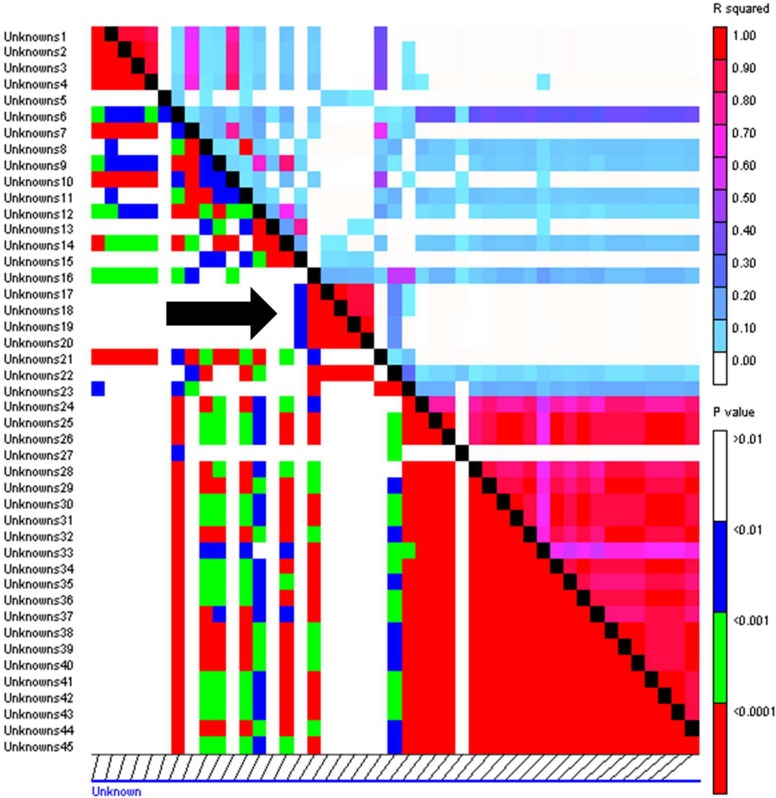
LD among markers on chromosome 3B. The four markers wPt-2193, wPt-2766, wPt-22988 and wPt-732330, associated with field crown score (FCS), are shown by an arrow in an LD block.

**Figure 8:**
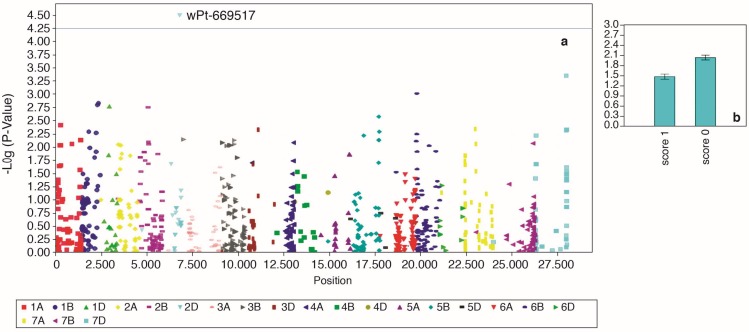
Manhattan plot showing association of 2D marker wPt-669517 with CR resistance using greenhouse stem scores (**a**) and allelic effect of wPt-669517 (**b**).

General linear model (GLM) and mixed linear model (MLM) were tested for all four traits and the best model for each was identified based on quantile-quantile (QQ) plot (Figure S2). In total, three marker-trait associations (MTAs) linked to CR resistance were identified which crossed false detection rate (FDR) at *p* < 0.05. For field crown score, GLM was the best model and four markers; wPt-2193, wPt-2766, wPt-22988 and wPt-732330 were identified to be associated with the trait (Figure 6). These four markers were, however, in an LD block (Figure 7) with high inter-marker *r*^2^ values (*r*^2^ = 0.93 to 0.99). Only two that passed the FDR test are shown in Table 3. The two markers (wPt-2193 and wPt-2766) were coincident at 39.1 cM on chromosome 3B. For greenhouse stem score, one marker wPt-669517, with a *p*-value of 3.24 × 10^−5^ (Table 3) was identified through MLM as being significantly linked to crown rot resistance (Figure 8). For growth room scores, neither GLM nor MLM were shown to be suitable models. The naïve model was the best for this trait (Figure S2). However, no significant markers were identified for growth room scores.

## 3. Discussion

CR is one of the most destructive wheat diseases, particularly under drought conditions [15]. Varying levels of success have been achieved using fungicides to control this disease. A recent study also investigated the effect of spraying speed (5, 8.5 or 12 km/h) on the occurrence of Fusarium head blight (FHB) and deoxynivalenol (DON) content in grains [36]. The spraying speed did not affect the DON content in the grains, although a significantly lower FHB incidence occurred at the 5 and 8.5 km/h spraying speeds. Chemical control therefore is sometimes effective but not economically feasible and environmentally suitable.

The use of resistant cultivars is one of the most effective strategies for reducing CR damage, but requires rapid, reliable, and reproducible CR screening assays. Various high-throughput screening methods have been used to identify novel sources of CR resistance in wheat [29,34,37,38,39,40,41,42,43], and a previous study investigated three different inoculation techniques (seedling dip, stem base droplet, and colonized grain) as potential methods [43]. The colonized grain method gave the best results, thus this method was also applied in the current study to minimize the variation often reported in CR screening tests. Standard check lines with known resistance to CR were included into the trails to provide greater certainty when comparing the results under different conditions.

Our study demonstrates significant genetic variation in the CR resistance responses of CIMMYT semi-arid spring bread wheat varieties under different environmental conditions. The highest disease infestation was found in growth room trials, compared to greenhouse and field conditions, as it has optimal conditions for CR development. Differences in reactions to *F. culmorum* under greenhouse and field conditions can be explained by climatic conditions, which play an important role in CR disease development [40]. Our study further confirmed that varieties 2–49 and Sunco (MR to *F. pseudograminearum*) are resistant to *F. culmorum*. Three spring bread wheat lines with synthetic hexaploid wheat backgrounds showed high levels of CR resistance especially at seedling level (Table 1). Another three spring bread wheat accessions BABAX/LR42//BABAX/3/BABAX/LR42//BABAX/4/T.DICOCCONPI94625/AE.SQUARROSA(372)//3*PASTOR/5/T.DICOCCON,PI94625/AE.SQUARROSA(372)//3*PASTOR, ACHTAR/4/MILAN/KAUZ//PRINIA/3/BAV92, and SOKOLL//FRTL/2*PIFED) showed high levels of resistance at both seedling and adult levels. Synthetic hexaploid wheat with CR resistance has previously been reported [44,45,46]; these lines can be utilized to develop CR-resistant lines.

Genotyping analysis showed that the distribution of polymorphic DArT markers differs among the A, B, and D genomes. Consistent with previous findings, the A and B genomes have higher polymorphism than D genome, which is attributed to higher number of effective recombinations in A and B genomes than D genome [19,47,48]. Structure analysis demonstrated the grouping of the panel in three groups. The partitioning of the genetic diversity in the subgroups is similar to what has been observed in other elite wheat collections [47,49]. The presence of non-random “background” co-ancestry among accessions is a common feature of cultivars and advanced breeding materials. This grouping is important for breeders to select lines for the breeding program.

The principle of GWAS is based on linkage disequilibrium (LD). The estimation of LD and more importantly the decay of LD governs the resolution of association mapping studies. LD decay over genetic distance in a population controls the density of maker coverage; a rapid decay suggests that a higher number of markers are required to carry out association mapping [48,50]. Our results showed that genome-wide LD decayed at approximately 20 cM. This value is similar to what has been reported in many previous studies in wheat [47,51,52,53,54]. A few recent studies [19,48,49,55,56,57,58], however, reported a faster LD decay (up to 5 or 10 cM), which points towards high genetic diversity of the panels reported in these studies compared to ours. The current panel had undergone intense selection pressure while selecting variability for resistance to crown rot and hence was compromised for genetic diversity.

GWAS is an effective method for identifying new QTL linked to agronomic traits and disease resistance [59,60,61,62,63], though it sometimes requires a considerably large population for accurate results. While we used the minimum acceptable size, the statistical approaches employed can detect the true signal of association analysis and are therefore reliable [50,64]. After controlling for population structure and family-relatedness, our study identified three MTAs for CR resistance. The two MTAs identified on chromosome 3B (wPt-2193 and wPt-2766), associated with field crown score (adult plant resistance), had exactly the same position on chromosome 3B and also were in high LD (Figure 7) and hence were part of the same QTL (Table 3). Previous studies have detected markers for crown rot resistance on 3BL [32,33,65]. Based on the consensus GBS, DArT and SSR map (DArT Pty Ltd. (http://www.diversityarrays.com/sequence-maps), wPt-2193 and wPt-2766 are located on short arm and hence likely represent a novel QTL for CR resistance. The other marker (wPt-669517), which is associated with greenhouse stem scores is positioned on the long arm of the chromosome 2D (DArT Pty Ltd.), which could be the same QTL as previously reported by [31,65,66].

Phenotypic disease assessment to determine wheat genetic resistance at the seedling and/or adult plant stage is currently time-consuming and labor intensive, requiring years to validate genotype resistance. CR is not been given enough attention in breeding programs even though it can cause significant damage to the wheat crop in terms of both quality and quantity. CR managements by other known cultural practices such as stubble burning and crop rotation have major limitations. Thus, the most effective way to control CR is by developing resistant wheat germplasm. Therefore, GWAS is a first step in identifying novel sources of CR resistance in CIMMYT germplasm. The three MTAs identified in this study can be used for screening for CR resistance and could be used to pyramid resistant loci through marker-assisted backcrossing.

## 4. Materials and Methods

### 4.1. Plant Materials

Test materials comprised 126 high yielding spring bread wheat lines adapted to both irrigated and non-irrigated conditions were phenotyped against CR, however, a set of 107 lines were genotyped and used in the GWAS (Table 1). These lines were developed by the CIMMYT spring bread wheat breeding program in Mexico. Accessions were selected based on their diverse genetic background and tested under growth room, greenhouse, and field conditions. Five susceptible and three resistant cultivars were used as controls (Table 1).

### 4.2. Inoculum Preparation

A monosporic isolate of *F. culmorum*, originally isolated from an infected wheat plant in Kırsehir, Turkey (39°39′709″ N, 34°25′515″ E), was transferred on synthetic nutrient agar and cultured at a temperature of 23 ± 1 °C with a 12 h photoperiod for 10 days for spore formation. Oven bags (35 cm × 48 cm), quarter filled with wheat bran, were humidified and sealed with cotton. The bags were autoclaved at 121 °C for 20 min over three successive days. Spore suspension was prepared by adding sterilized distilled water to each petri dish containing *F. culmorum* culture. Bags of autoclaved wheat bran were left to cool and then inoculated with the spore suspension under sterilized conditions. Inoculated wheat bran was mixed by shaking the bags and incubated at a temperature of 23 ± 1 °C for 2–3 weeks with a 12 h photoperiod until the bran was sufficiently colonized by the fungus. Finally, fungus-colonized wheat bran was left to dry at room temperature. The *F. culmorum* inoculum was used for all three experiments (growth room, greenhouse, and field trials).

### 4.3. Fusarium culmorum Disease Screening

#### 4.3.1. Growth Room

Wheat bran inoculated with the fungus was suspended in distilled water and filtered through two layers of cheesecloth. Spore concentration was adjusted to 1 × 10^6^ spores mL^−1^, and methyl cellulose (0.1%) was added to the spore suspension prior to use. Ten wheat seeds were placed on moist blotting paper in sterilized petri dishes for germination at 22 °C for 2–3 days to obtain sufficient plantlets with a similar phenological stage. Each pre-germinated seed was sown in a different plastic tube (2.5 cm diameter × 16 cm height; Ray Leach Container, Canby, OR, USA) filled with potting mix (62 g) and covered with the same substrate. A sterile potting mix of sand, soil, and organic manure (50:40:10; *v*/*v*/*v*) was used for growth room and greenhouse trials. One week after sowing, the stem base of each seedling (0.5–1 cm above the soil level, including the coleoptile) was inoculated with *F. culmorum* at a concentration of 1 × 10^6^ spores mL^−1^ of the spore suspension. Inoculated tubes were covered with plastic sheeting to maintain high humidity (80–90%) and kept at 23 ± 1 °C for 48 h. After incubation, seedlings were kept in a growth room for 42 days (early tillering, Zadoks growth stage 14 [62]), with a day/night photoperiod of 16/8 h, at 23 ± 1 °C, and relative humidity of 60/80% (±5%). Randomized complete block design (RCBD) with five replications was used (1 plant per replicate) and repeated twice.

#### 4.3.2. Greenhouse

Two seeds of each wheat accession were grown in each tube, along with 0.5 g fungus-colonized wheat bran (as an inoculum source). Tubes were then placed in a stand on sand in the greenhouse to facilitate root growth. Experiments were watered as required during the growing season. To promote disease development, plants were subjected to water stress at maturity stages. The experiment was set up using a RCBD with six replications (two plants per replicate).

#### 4.3.3. Field Conditions

Plant materials and check cultivars were planted at the Ilci Agricultural Research Institute, Yozgat, Turkey (Latitude 39.63806; Longitude 34.46722) under naturally infested field conditions during the 2011/12 growing season (October to June). A 5 g seed sample of each entry was sown in a 1 m row and infected with 2 g of fungus-colonized wheat bran. Experiments were arranged using a RCBD with three replications. Disease symptoms were scored by picking 15 individual plants from each row.

### 4.4. Disease Assessment and Data Analysis

At the end of the growing period, plants were harvested and stems were collected. Plants grown under growth room conditions were evaluated for seedling resistance (Zadoks growth stage 14). Plants grown under greenhouse and field conditions were evaluated at maturity and tested for adult plant resistance. Plants were scored for the typical symptoms of browning percentage on the crown (by observing the disease on the crown of the plants) and the main stem (by measuring the disease symptoms on the stem) using numeric scales (1–5) modified from [31]: resistant (1: 1–9%), moderately resistant (2: 10–29%), moderately susceptible (3: 30–69%), susceptible (4: 70–89%), and highly susceptible (5: 90–99%). Plants grown under field conditions were also scored for white head symptoms at the ripening stage (Zadoks growth stage 91–94) using a 1–5 scale. Data were analyzed using the standard analysis of variance procedure in the GenStat 14 program for windows (VSN international, http://www.vsni.co.uk/software/genstat). Differences between growing conditions were investigated using an LSD test, with statistical differences considered significant at *p* ≤ 0.05.

### 4.5. Genotyping with DArT Markers

Genomic DNA was extracted from a bulk of 10 two-week old seedling leaves using a modified cetyltrimethylammonium bromide procedure [67,68], in accordance with CIMMYT protocols [69]. All 107 accessions were genotyped with 1726 DArT markers [65]. Monomorphic markers and those with missing marker data greater than 20% with minor allele frequency <0.05 and >0.95, were culled.

### 4.6. Population Structure Analysis

Population structure of the 107 genotypes was analyzed using the Bayesian clustering method implemented in STRUCTURE software (version 5.0) to identify clusters of genetically similar individuals [70,71,72,73]. To minimize the effect of starting configuration, a burn-in length of 10^4^ cycles was applied, followed by a simulation run of 10^6^ cycles using the admixture model option in Structure. Cluster values (K) ranging from 2 to 24 were chosen, with six independent runs conducted for each value. A zip archive containing all of the results-f files was created and used as an input in Structure Harvester program to estimate delta K (∆K). It is an ad hoc measure, which identifies the number of subpopulations by estimating the rate of change in the log probability of data between successive K values. The principal component analysis (PCA) was performed the function PRCOMP from the STATS package in R.

### 4.7. Linkage Disequilibrium and GWAS

Linkage disequilibrium (LD) analysis was performed using polymorphic markers with allele frequency higher than 5% using Trait Analysis by Association, Evolution, and Linkage (TASSEL) software (version V5.0) [74,75]. LD was estimated as squared allele frequency correlations (*r*^2^) and, for each pair of loci, only *p*-values ≤ 0.01 were considered significant. To identify the QTL linked to CR resistance, GWAS was conducted in TASSEL for the adult-plant resistance scores (GHSS, GHCS, FCS) using the average value of all the replications. For the seedling resistance score (GRCS), the frequency of resistant lines was too low to consider in GWAS. Two models, GLM (with population structure) and MLM (with population structure and K-matrix), were tested for all traits and the best model was selected by drawing QQ plots. The naïve model (no control for population structure and relatedness) was tested only if none of the GLM or MLM models were found suitable. False discovery rate values were calculated at 0.05 based on Benjamini and Hochberg (1995) [76].

## Figures and Tables

**Table 1:** Arithmetic means of 12 crown rot resistant spring bread wheat lines assessed under growth room conditions (seedling resistance) or greenhouse and field conditions (adult plant resistance), compared to eight standard checks.

Cross Name	CID	Adult-Plant Resistance			Seedling Resistance	
		GHSS	GHCS	FCS	GRCS	RR
BABAX/LR42//BABAX/3/BABAX/LR42//BABAX/4/T.DICOCCON PI94625/AE.SQUARROSA (372)//3*PASTOR/5/T.DICOCCON PI94625/AE.SQUARROSA (372)//3*PASTOR	6,000,229	2.2	2.5	2.2	2.5	A-S
BABAX/LR42//BABAX/3/BABAX/LR42//BABAX/4/T.DICOCCON PI94625/AE.SQUARROSA (372)//3*PASTOR/5/T.DICOCCON PI94625/AE.SQUARROSA (372)//3*PASTOR	6,000,238	1.5	2.2	2.7	2.2	S
FRET2*2/4/SNI/TRAP#1/3/KAUZ*2/TRAP//KAUZ/5/ONIX	6,000,365	1.5	2.5	3	1.8	S
ACHTAR/4/MILAN/KAUZ//PRINIA/3/BAV92	6,000,537	2.2	2.5	2.5	2.3	A-S
SOKOLL*2/ROLF07	6,000,973	1.5	2.5	2.3	3.2	A
GK ARON/AG SECO 7846//2180/4/2*MILAN/KAUZ//PRINIA/3/BAV92	6,001,016	2.5	3.5	3.2	1.8	S
SOKOLL//FRTL/2*PIFED	6,001,172	2.2	2.8	2.5	2.3	A-S
ROLF07/3/T.DICOCCON PI94625/AE.SQUARROSA (372)//3*PASTOR	6,001,240	2.2	2.8	3	2.3	S
CUNNINGHAM/4/SNI/TRAP#1/3/KAUZ*2/TRAP//KAUZ	6,001,457	2.2	2.8	3	1.8	S
SOKOLL*2/4/CHEN/AEGILOPS SQUARROSA (TAUS)//FCT/3/STAR	6,001,643	2.5	3.5	2.7	2.3	S
SERI*3//RL6010/4*YR/3/PASTOR/4/BAV92/5/MONARCA F2007/6/PVN//CAR422/ANA/5/BOW/CROW//BUC/PVN/3/YR/4/TRAP#1	6,000,104	2.2	2.3	2.5	2.8	A
CNO79//PF70354/MUS/3/PASTOR/4/BAV92/5/FRET2/KUKUNA//FRET2/6/MILAN/KAUZ//PRINIA/3/BAV92	6,000,615	1.5	2.8	3.5	2.3	S
2–49		1.3	2.3	2.5	2.5	MR
Altay		1.2	2.5	2.3	2.4	MR
Sunco		1.5	2.5	2.3	2.6	MR
Seri		2.2	3.2	3.5	4	MS
Kiziltan		2.5	3.5	4	4	HS
Kutluk		2.5	3.5	4	4	HS
Wylie		2.3	3.3	3.4	3.5	S
Janz		2.5	3.5	3.7	4	HS
LSD		0.5	0.53	1.04	1.08	
Probability		<0.001	<0.001	<0.001	<0.001	

Abbreviations stand for: ID = cross identification; GHSS = greenhouse stem score; GHCS = greenhouse crown score; FCS = field crown score; GRCS = growth room crown score; RR = resistance reaction; A = adult resistance; S = seedling resistance; LSD = least significant difference. 1–5 scale modified from Wildermuth and McNamara (1994) (resistant = R, 1: 1–9%; moderately resistant = MR, 2: 10–29%; moderately susceptible = MS, 3: 30–69%; susceptible = S, 4: 70–89%; and highly susceptible = HS, 5: 90–99%) [31]. Means = backcross, A/*2B = A/B//B − backcross, A/*3b = A/B//B/3/B − 2 backcross, # means number.

**Table 2:** Correlation among the different traits studied. The above diagonal values represent Pearson’s correlation values and below diagonal values represent *p* values.

	GHCS	FCR	GRCS	GHSS
GHCS	1.00000	0.10153	0.01128	**0.50728**
FCR	0.2388	1.00000	**0.31369**	0.14685
GRCS	0.89671	**0.00010**	1.00000	0.13237
GHSS	**0.00000**	0.08574	0.12258	1.00000

Abbreviations stand for: GHSS = greenhouse stem score; GHCS = greenhouse crown score; FCS = field crown score; GRCS = growth room crown score. Above diagonal bold values represent correlations that were higher than most others and lower diagonal bold values represent level of significance of the corresponding correlations.

**Table 3:** Marker trait associations for crown rot resistance using general linear model (including on Q matrix) and mixed linear model (Q + kinship matrices). Only markers that crossed FDR adjustment are shown here. FCS = Field crown score and GHSS = Greenhouse stem score.

Trait	Marker	CHR	POS (cM)	*p*-Value	FDR Adjusted *p*-Values	*r*^2^ (%)
FCS (GLM)	wPt-2193	3B	39.1	6.56 × 10^−5^	0.01232	11.4
FCS (GLM)	wPt-2766	3B	39.1	6.56 × 10^−5^	0.01232	11.4
GHSS (MLM)	wPt-669517	2D	95.7	3.24 × 10^−5^	0.01997	11.6

## Supplementary Materials

Supplementary materials are available online at http://www.mdpi.com/1422-0067/19/9/2666/s1.
