# Supplementary material for: Identification of Novel Quantitative Trait Loci Linked to Crown Rot Resistance in Spring Wheat
Source: Int J Mol Sci. 2018 Sep 8;19(9):2666. doi: 10.3390/ijms19092666 (PMC6165080; doi:10.3390/ijms19092666)
Supplement: Supplementary file 1 [file ijms-19-02666-s001.pdf]

Supplementary Materials

Identification of Novel Quantitative Trait Loci Linked to Crown Rot Resistance in Spring Wheat

Table S1. List of 126 spring wheat accessions with accession number, common name, selection history, origin, and host response to crown rot. List of 126 CIMMYT advanced spring wheat lines from the 29th Semi-Arid Wheat Screening Nursery (29th SAWSN) with accession cross name, germplasm identification (GID), cross identification (CID), selection identification (SID), phenotyped (P) against *Fusarium culmorum* along with their response to crown rot (1–5 scale) under the three different environmental conditions (Growth room, Greenhouse, and Field), and/or genotyped (G) for the association mapping in this study.

#	Cross name	CID	SID	GID	Response to *Fusarium culmorum* (CR score)			P/G
					Growth room	Greenhouse	Field	
1	POTCH 93/4/MILAN/KAUZ//PRINIA/3/BAV92/5/MILAN/KAUZ//PRINIA/3/BAV92	512755	69	5999970	3	2	3	PG
2	ACHTAR*3//KANZ/KS85-8-5/4/MILAN/KAUZ//PRINIA/3/BAV92/5/MILAN/KAUZ//PRINIA/3/BAV92	512797	154	5999989	3	3	3	PG
3	QG 4.37A/4/MILAN/KAUZ//PRINIA/3/BAV92/5/MILAN/KAUZ//PRINIA/3/BAV92	512844	121	6000027	3	2	3	PG
4	NSM*4/14-2//FRTL/2*PIFED/3/VORB	512786	100	6000219	3	2	3	PG
5	BABAX/LR42//BABAX/3/BABAX/LR42//BABAX/4/T.DICOCCON PI94625/AE.SQUARROSA (372)//3*PASTOR/5/T.DICOCCON PI94625/AE.SQUARROSA (372)//3*PASTOR	512908	124	6000229	2	2	2	PG

6	BABAX/LR42//BABAX/3/BABAX/LR42//BABAX/4/T.DICOCCON PI94625/AE.SQUARROSA (372)//3*PASTOR/5/T.DICOCCON PI94625/AE.SQUARROSA (372)//3*PASTOR	512908	133	6000238	2	2	3	PG
7	FRET2*2/4/SNI/TRAP#1/3/KAUZ*2/TRAP//KAUZ/5/ONIX	504237	155	6000355	2	2	3	PG
8	FRET2*2/4/SNI/TRAP#1/3/KAUZ*2/TRAP//KAUZ/5/ONIX	504237	165	6000365	2	2	3	PG
9	ONIX/ROLF07	504248	70	6000384	3	2	3	PG
10	ONIX/ROLF07	504248	76	6000390	3	2	3	PG
11	ONIX/4/MILAN/KAUZ//PRINIA/3/BAV92	504250	113	6000404	4	2	4	PG
12	ACHTAR/4/MILAN/KAUZ//PRINIA/3/BAV92	504457	64	6000537	2	2	3	PG
13	CNO79//PF70354/MUS/3/PASTOR/4/BAV92/5/FRET2/KUKUNA//FRET2/6/MILAN/KAUZ//PRINIA/3/BAV92	506887	98	6000614	3	3	3	PG
14	CNO79//PF70354/MUS/3/PASTOR/4/BAV92/5/FRET2/KUKUNA//FRET2/6/MILAN/KAUZ//PRINIA/3/BAV92	506887	101	6000617	3	3	2	PG
15	CNO79//PF70354/MUS/3/PASTOR/4/BAV92/5/FRET2/KUKUNA//FRET2/6/MILAN/KAUZ//PRINIA/3/BAV92	506887	103	6000619	3	2	3	PG
16	CNO79//PF70354/MUS/3/PASTOR/4/BAV92/5/FRET2/KUKUNA//FRET2/6/MILAN/KAUZ//PRINIA/3/BAV92	506887	107	6000623	4	3	3	PG
17	MILAN/KAUZ//PRINIA/3/BAV92/4/ATTILA/BAV92//PASTOR/5/CNO79//PF70354/MUS/3/PASTOR/4/BAV92	506898	48	6000648	4	1	3	PG
18	SOKOLL*2/TROST	507062	97	6000906	4	3	3	PG
19	SOKOLL*2/TROST	507062	100	6000909	4	3	3	PG
20	SOKOLL//PBW343*2/KUKUNA/3/ATTILA/PASTOR	507064	86	6000917	4	2	3	PG
21	SOKOLL*2/ROLF07	507102	57	6000973	3	2	2	PG
22	GK ARON/AG SECO 7846//2180/4/2*MILAN/KAUZ//PRINIA/3/BAV92	506830	98	6001013	2	3	4	PG

23	GK ARON/AG SECO 7846//2180/4/2*MILAN/KAUZ//PRINIA/3/BAV92	506830	99	6001014	3	3	3	PG
24	GK ARON/AG SECO 7846//2180/4/2*MILAN/KAUZ//PRINIA/3/BAV92	506830	101	6001016	2	3	3	PG
25	SW89-5124*2/FASAN/3/ALTAR 84/AE.SQ//2*OPATA	495230	91	6001072	3	3	3	PG
26	SOKOLL/ROLF07	495241	91	6001093	3	3	3	PG
27	SOKOLL//FRTL/2*PIFED	495447	74	6001172	2	3	3	PG
28	SOKOLL//FRTL/2*PIFED	495447	81	6001179	3	3	3	PG
29	BAV92/SERI	285851	1382	6001235	3	3	3	PG
30	ROLF07/3/T.DICOCCON PI94625/AE.SQUARROSA (372)//3*PASTOR	504090	84	6001239	4	3	3	PG
31	ROLF07/3/T.DICOCCON PI94625/AE.SQUARROSA (372)//3*PASTOR	504090	85	6001240	2	3	3	PG
32	MILAN/KAUZ//PRINIA/3/BAV92/4/WBLL1*2/KUKUNA	494935	32	6001357	4	3	3	PG
33	ATTILA/BAV92//PASTOR/3/ATTILA*2/PBW65	494965	49	6001439	4	3	4	PG
34	CUNNINGHAM/4/SNI/TRAP#1/3/KAUZ*2/TRAP//KAUZ	494983	41	6001457	2	3	3	PG
35	ESDA/KKTS	495073	28	6001472	3	3	4	PG
36	GOUBARA-1/2*SOKOLL	495915	79	6001556	3	3	3	PG
37	SOKOLL*2/3/PASTOR//MUNIA/ALTAR 84	496026	97	6001639	4	3	3	PG
38	SOKOLL*2/3/PASTOR//MUNIA/ALTAR 84	496026	99	6001641	3	3	2	PG
39	SOKOLL*2/4/CHEN/AEGILOPS SQUARROSA (TAUS)//FCT/3/STAR	496027	103	6001643	2	3	3	PG
40	SOKOLL*2/4/CHEN/AEGILOPS SQUARROSA (TAUS)//FCT/3/STAR	496027	105	6001645	4	3	3	PG
41	BOW/VEE/5/ND/VG9144//KAL/BB/3/YACO/4/CHIL/6/CASKOR/3/CROC_1/AE.SQUARROSA (224)//OPATA/7/PASTOR//MILAN/KAUZ/3/BAV92	496096	35	6001669	4	3	3	PG

42	BOW/VEE/5/ND/VG9144//KAL/BB/3/YACO/4/CHIL/6/CASKOR/3/CROC_1/AE.SQUARROSA (224)//OPATA/7/PASTOR//MILAN/KAUZ/3/BAV92	496096	39	6001673	4	3	3	PG
43	BOW/VEE/5/ND/VG9144//KAL/BB/3/YACO/4/CHIL/6/CASKOR/3/CROC_1/AE.SQUARROSA (224)//OPATA/7/PASTOR//MILAN/KAUZ/3/BAV92	496096	41	6001675	4	3	4	PG
44	GONDO/MONARCA F2007/4/GONDO//SHA5/WEAVER/3/PASTOR	512743	133	6001847	4	3	4	PG
45	PASTOR*2/BAV92/5/FRET2*2/4/SNI/TRAP#1/3/KAUZ*2/TRAP//KAUZ	494961	50	6001910	4	3	4	PG
46	PASTOR*2/BAV92/5/FRET2*2/4/SNI/TRAP#1/3/KAUZ*2/TRAP//KAUZ	494961	52	6001912	4	3	3	PG
47	BABAX/LR39//BABAX/3/FILIN/2*PASTOR	512108	54	5999744	4	3	3	PG
48	YAR/AE.SQUARROSA (518)/3/PRL/SARA//TSI/VEE#5/4/ATTILA/5/BERKUT	512186	69	5999851	4	2	3	PG
49	YAR/AE.SQUARROSA (518)/3/PRL/SARA//TSI/VEE#5/4/ATTILA/5/BERKUT	512186	72	5999854	4	3	3	PG
50	ACHTAR*3//KANZ/KS85-8-5/4/MILAN/KAUZ//PRINIA/3/BAV92/5/MILAN/KAUZ//PRINIA/3/BAV92	512797	148	5999983	4	3	3	PG
51	ACHTAR*3//KANZ/KS85-8-5/4/MILAN/KAUZ//PRINIA/3/BAV92/5/MILAN/KAUZ//PRINIA/3/BAV92	512797	149	5999984	4	3	3	PG
52	QG 4.37A/4/MILAN/KAUZ//PRINIA/3/BAV92/5/MILAN/KAUZ//PRINIA/3/BAV92	512844	141	6000047	4	3	3	PG
53	QG 78.5//2*INQALAB 91*2/TUKURU	512849	146	6000050	3	3	2	PG
54	QG 78.5//2*INQALAB 91*2/TUKURU	512849	163	6000067	3	3	3	PG
55	EGA BONNIE ROCK*2/5/FRET2*2/4/SNI/TRAP#1/3/KAUZ*2/TRAP//KAUZ	512870	86	6000083	3	2	3	PG

56	EGA BONNIE ROCK*2/5/FRET2*2/4/SNI/TRAP#1/3/KAUZ*2/TRAP//KAUZ	512870	88	6000085	3	3	3	PG
57	HUANIL//2*WBLL1*2/KUKUNA	512888	102	6000092	4	3	3	PG
58	SERI*3//RL6010/4*YR/3/PASTOR/4/BAV92/5/MONARCA F2007/6/PVN//CAR422/ANA/5/BOW/CROW//BUC/PVN/3/YR/4/TRAP#1	512926	130	6000104	3	2	3	PG
59	SERI*3//RL6010/4*YR/3/PASTOR/4/BAV92/5/MONARCA F2007/6/PVN//CAR422/ANA/5/BOW/CROW//BUC/PVN/3/YR/4/TRAP#1	512926	133	6000107	3	3	3	PG
60	SERI*3//RL6010/4*YR/3/PASTOR/4/BAV92/5/MONARCA F2007/6/PVN//CAR422/ANA/5/BOW/CROW//BUC/PVN/3/YR/4/TRAP#1	512926	149	6000123	4	2	3	PG
61	BABAX/LR42//BABAX/3/BABAX/LR42//BABAX/4/T.DICOCCON PI94625/AE.SQUARROSA (372)//3*PASTOR/5/T.DICOCCON PI94625/AE.SQUARROSA (372)//3*PASTOR	512908	122	6000227	4	3	2	PG
62	BABAX/LR42//BABAX/3/BABAX/LR42//BABAX/4/T.DICOCCON PI94625/AE.SQUARROSA (372)//3*PASTOR/5/T.DICOCCON PI94625/AE.SQUARROSA (372)//3*PASTOR	512908	125	6000230	3	2	3	PG
63	BABAX/LR42//BABAX/3/BABAX/LR42//BABAX/4/T.DICOCCON PI94625/AE.SQUARROSA (372)//3*PASTOR/5/T.DICOCCON PI94625/AE.SQUARROSA (372)//3*PASTOR	512908	129	6000234	4	3	3	PG
64	BABAX/LR42//BABAX/3/BABAX/LR42//BABAX/4/T.DICOCCON PI94625/AE.SQUARROSA (372)//3*PASTOR/5/T.DICOCCON PI94625/AE.SQUARROSA (372)//3*PASTOR	512908	135	6000240	3	3	2	PG
65	MILAN/KAUZ//PRINIA/3/BAV92/5/TRAP#1/BOW//VEE#5/SARA/3/ZHE JIANG 4/4/DUCULA	504111	152	6000264	4	3	4	PG

66	MILAN/KAUZ//PRINIA/3/BAV92/5/TRAP#1/BOW//VEE#5/SARA/3/ZHE JIANG 4/4/DUCULA	504111	157	6000269	4	3	3	PG
67	FRET2*2/4/SNI/TRAP#1/3/KAUZ*2/TRAP//KAUZ/5/ONIX	504237	153	6000353	4	3	4	PG
68	FRET2*2/4/SNI/TRAP#1/3/KAUZ*2/TRAP//KAUZ/5/ONIX	504237	159	6000359	3	2	3	PG
69	FRET2*2/4/SNI/TRAP#1/3/KAUZ*2/TRAP//KAUZ/5/ONIX	504237	163	6000363	3	3	3	PG
70	FRET2*2/4/SNI/TRAP#1/3/KAUZ*2/TRAP//KAUZ/5/ONIX	504237	168	6000368	3	2	4	PG
71	ONIX/ROLF07	504248	73	6000387	4	3	3	PG
72	ONIX/4/MILAN/KAUZ//PRINIA/3/BAV92	504250	105	6000396	3	3	3	PG
73	ONIX/4/MILAN/KAUZ//PRINIA/3/BAV92	504250	108	6000399	4	3	3	PG
74	BARCENAS S2002/4/MILAN/KAUZ//PRINIA/3/BAV92	504493	54	6000547	3	2	3	PG
75	FDC36//ATTILA*2/PBW65	504539	32	6000566	4	2	3	PG
76	CNO79//PF70354/MUS/3/PASTOR/4/BAV92/5/ATTILA*2/PBW65/6/PBW343*2/TUKURU	506892	45	6000630	4	2	3	PG
77	RL6043/4*NAC//PASTOR/3/BAV92/4/ATTILA/PASTOR/5/PBW343*2/TUKURU	506911	81	6000684	4	2	3	PG
78	SOKOLL//PBW343*2/KUKUNA/3/ATTILA/PASTOR	507064	91	6000922	4	3	3	PG
79	SW89-5124*2/FASAN/3/ALTAR 84/AE.SQ//2*OPATA/4/ARREHANE	507096	42	6000940	3	2	2	PG
80	SOKOLL*2/ROLF07	507102	54	6000970	3	3	3	PG
81	CNDO/R143//ENTE/MEXI_2/3/AEGILOPS SQUARROSA (TAUS)/4/WEAVER/5/2*JANZ*2/6/BORL95/3*JANZ	507180	125	6000993	4	2	3	PG
82	SOKOLL//ATTILA/PASTOR/3/PBW343*2/TUKURU	507236	94	6000999	4	3	4	PG
83	SOKOLL//ATTILA/PASTOR/3/PBW343*2/TUKURU	507236	101	6001006	4	3	3	PG
84	TROST/6/CNDO/R143//ENTE/MEXI_2/3/AEGILOPS SQUARROSA (TAUS)/4/WEAVER/5/PASTOR	495132	75	6001043	4	3	4	PG
85	TROST/6/CNDO/R143//ENTE/MEXI_2/3/AEGILOPS SQUARROSA (TAUS)/4/WEAVER/5/PASTOR	495132	77	6001045	4	3	3	PG

86	SOKOLL/TROST	495180	79	6001056	4	3	3	PG
87	SOKOLL/TRCH	495192	163	6001066	3	2	2	PG
88	SW89-5124*2/FASAN/3/ALTAR 84/AE.SQ//2*OPATA	495230	93	6001074	4	1	3	PG
89	SOKOLL/ROLF07	495241	85	6001087	3	2	2	PG
90	SOKOLL/ROLF07	495241	87	6001089	3	2	3	PG
91	SOKOLL/ROLF07	495241	89	6001091	4	2	3	PG
92	CNDO/R143//ENTE/MEXI_2/3/AEGILOPS SQUARROSA (TAUS)/4/WEAVER/5/2*JANZ/6/SOKOLL	495362	113	6001124	4	2	3	PG
93	D67.2/PARANA 66.270//AE.SQUARROSA (320)/3/CUNNINGHAM/4/WBLL1*2/TUKURU	495387	100	6001137	4	2	3	PG
94	D67.2/PARANA 66.270//AE.SQUARROSA (320)/3/CUNNINGHAM/4/WBLL1*2/TUKURU	495387	101	6001138	3	3	3	PG
95	ROLF07/3/T.DICOCCON PI94625/AE.SQUARROSA (372)//3*PASTOR	504090	86	6001241	4	3	3	PG
96	T.DICOCCON PI94625/AE.SQUARROSA (372)//3*PASTOR/3/PBW343*2/KUKUNA/4/PBW343*2/KUKUNA	506652	48	6001247	4	3	4	PG
97	RL6043/4*NAC//PASTOR/3/BAV92/4/ATTILA/BAV92//PASTOR	494951	79	6001392	3	2	3	PG
98	PASTOR*2/BAV92/3/FRET2/KUKUNA//FRET2	494962	59	6001418	4	2	4	PG
99	PASTOR*2/BAV92/3/FRET2/KUKUNA//FRET2	494962	61	6001420	3	2	3	PG
100	GOUBARA-1/2*SOKOLL	495915	71	6001548	3	2	3	PG
101	GOUBARA-1/2*SOKOLL	495915	72	6001549	4	2	3	PG
102	GOUBARA-1/2*SOKOLL	495915	75	6001552	3	2	3	PG

103	PSN/BOW//MILAN/3/2*PARUS/PASTOR	495988	15	6001604	4	2	3	PG
104	SOKOLL*2/4/CHEN/AEGILOPS SQUARROSA (TAUS)//FCT/3/STAR	496027	102	6001642	3	3	3	PG
105	FRET2*2/4/SNI/TRAP#1/3/KAUZ*2/TRAP//KAUZ/5/ONIX	504237	170	6000370	4	2	3	PG
106	PASTOR*2/BAV92/5/FRET2*2/4/SNI/TRAP#1/3/KAUZ*2/TRAP//KAUZ	494961	51	6001911	4	2	3	PG
107	TUKURU/4/CROC_1/AE.SQUARROSA (224)//YACO/3/MUNIA/5/BABAX/LR42//BABAX	496164	110	6001966	3	3	3	PG
108	W15.92/4/PASTOR//HXL7573/2*BAU/3/WBLL1	473251	30	5435924	4	2	3	p
109	BABAX/LR39//BABAX/3/FILIN/2*PASTOR	512108	55	5999745	4	2	3	p
110	QG 78.5//2*INQALAB 91*2/TUKURU	512849	158	6000062	3	2	3	p
111	QG 78.5//2*INQALAB 91*2/TUKURU	512849	160	6000064	3	2	3	p
112	HUANIL//2*WBLL1*2/KUKUNA	512888	103	6000093	4	2	3	p
113	CNO79//PF70354/MUS/3/PASTOR/4/BAV92/5/FRET2/KUKUNA//FRET2/6/MILAN/KAUZ//PRINIA/3/BAV92	506887	99	6000615	2	2	4	p
114	CNO79//PF70354/MUS/3/PASTOR/4/BAV92/5/FRET2/KUKUNA//FRET2/6/MILAN/KAUZ//PRINIA/3/BAV92	506887	100	6000616	3	2	3	p
115	CNO79//PF70354/MUS/3/PASTOR/4/BAV92/5/FRET2/KUKUNA//FRET2/6/MILAN/KAUZ//PRINIA/3/BAV92	506887	102	6000618	3	2	3	p

116	CNO79//PF70354/MUS/3/PASTOR/4/BAV92/5/FRET2/KUKUNA//FRET2/6/MILAN/KAUZ//PRINIA/3/BAV92	506887	106	6000622	4	3	4	p
117	CNO79//PF70354/MUS/3/PASTOR/4/BAV92/5/FRET2/KUKUNA//FRET2/6/MILAN/KAUZ//PRINIA/3/BAV92	506887	108	6000624	4	3	3	p
118	CNO79//PF70354/MUS/3/PASTOR/4/BAV92/5/FRET2/KUKUNA//FRET2/6/MILAN/KAUZ//PRINIA/3/BAV92	506887	109	6000625	4	3	4	p
119	CNO79//PF70354/MUS/3/PASTOR/4/BAV92/5/FRET2/KUKUNA//FRET2/6/MILAN/KAUZ//PRINIA/3/BAV92	506887	112	6000628	4	3	4	p
120	MILAN/KAUZ//PRINIA/3/BAV92/4/PASTOR*2/BAV92/5/ROLF07	506897	36	6000640	4	2	3	p
121	SOKOLL//PBW343*2/KUKUNA/3/ATTILA/PASTOR	507064	90	6000921	4	2	3	p
122	GK ARON/AG SECO 7846//2180/4/2*MILAN/KAUZ//PRINIA/3/BAV92	506830	97	6001012	3	2	3	p
123	SOKOLL/TRCH	495192	161	6001064	3	2	3	p
124	CNDO/R143//ENTE/MEXI_2/3/AEGILOPS SQUARROSA (TAUS)/4/WEAVER/5/2*JANZ/6/SOKOLL	495362	115	6001126	3	1	4	P
125	BAV92/SERI	285851	1380	6001233	2	2	4	P
126	ESDA/KKTS	495073	27	6001471	4	2	4	P

Table S2. **Summary of linkage disequilibrium across chromosomes and sub-genomes.**

Chromosome*	Total number of marker pairs	% $r^2 \geq 0.1$	Average LD at $r^2 \geq 0.1$
1A	4025	37.3	0.40
1B	3225	52.8	0.47
1D	253	20.9	0.29
2A	1425	39.7	0.57
2B	2225	31.8	0.28
2D	253	42.6	0.35
3A	1375	38.6	0.37
3B	4475	27.7	0.43
3D	1375	69.6	0.65
4A	1275	50.2	0.29
4B	136	19.1	0.36
5A	171	29.8	0.36
5B	2025	25.4	0.33
6A	3825	32.7	0.34
6B	2975	25.2	0.37
6D	91	45.0	0.49
7A	2025	25.4	0.31
7B	1825	44.1	0.33
7D	3675	70.9	0.67

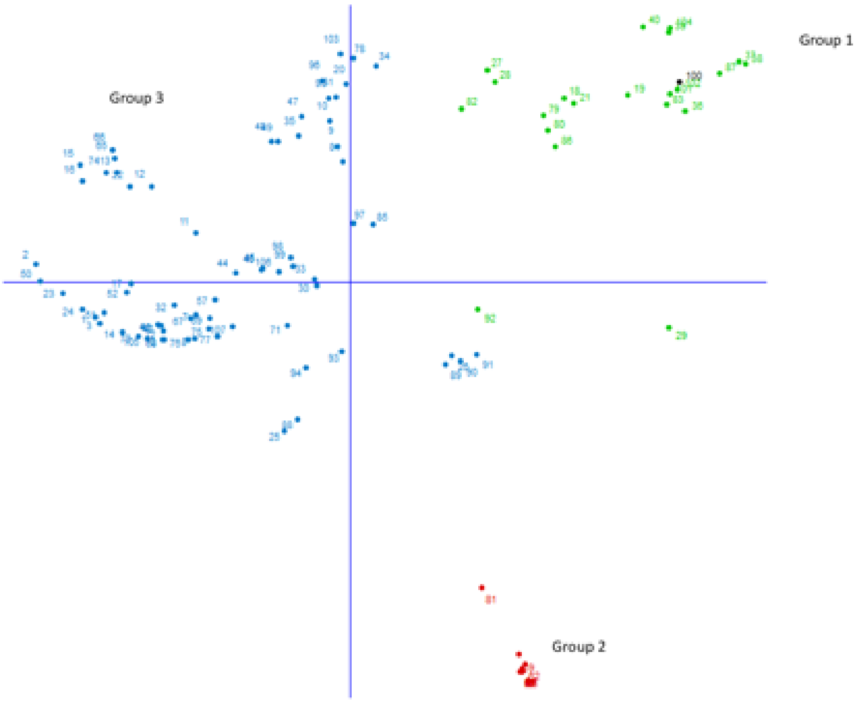

Figure S1. Principal component analysis of the 107 advanced CIMMYT spring wheat accessions of constructed with 1174 DArT markers. The PCs 1 and 3 are shown here.

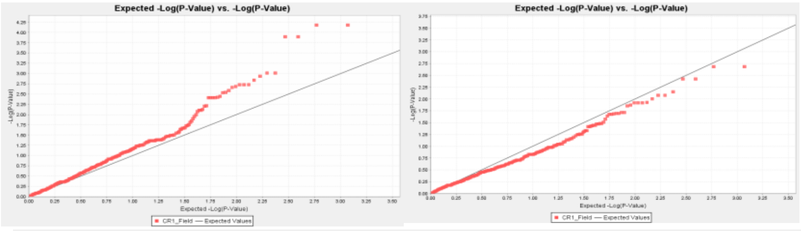

a) Field crown scores

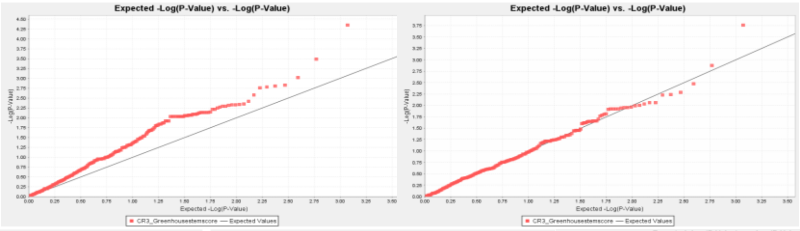

b) Greenhouse stem scores

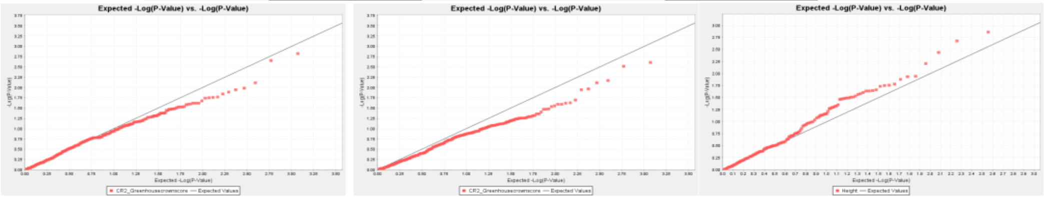

c) Growth room crown scores

Figure S2. **QQ plots for GLM and MLM models for Field crown scores (a), Greenhouse stem sco.**
